# Analysis of the Release Characteristics of Cu-Treated Antimicrobial Implant Surfaces Using Atomic Absorption Spectrometry

**DOI:** 10.1155/2012/850390

**Published:** 2011-11-24

**Authors:** Carmen Zietz, Andreas Fritsche, Birgit Finke, Vitezslav Stranak, Maximilian Haenle, Rainer Hippler, Wolfram Mittelmeier, Rainer Bader

**Affiliations:** ^1^Biomechanics and Implant Technology Research Laboratory, Department of Orhtopeadics, University of Rostock, Doberaner Stra*β*e 142, 18057 Rostock, Germany; ^2^Leibniz Institute for Plasma Science and Technology (INP e.V. Greifswald), Felix-Hausdorff-Stra*β*e 2, 17489 Greifswald, Germany; ^3^Institute of Physics, Ernst-Moritz-Arndt University of Greifswald, Felix-Hausdorff-Stra*β*e 6, 17487 Greifswald, Germany

## Abstract

New developments of antimicrobial implant surfaces doped with copper (Cu) ions may minimize the risk of implant-associated infections. However, experimental evaluation of the Cu release is influenced by various test parameters. The aim of our study was to evaluate the Cu release characteristics *in vitro* according to the storage fluid and surface roughness. Plasma immersion ion implantation of Cu (Cu-PIII) and pulsed magnetron sputtering process of a titanium copper film (Ti-Cu) were applied to titanium alloy (Ti6Al4V) samples with different surface finishing of the implant material (polished, hydroxyapatite and corundum blasted). The samples were submersed into either double-distilled water, human serum, or cell culture medium. Subsequently, the Cu concentration in the supernatant was measured using atomic absorption spectrometry. The test fluid as well as the surface roughness can alter the Cu release significantly, whereby the highest Cu release was determined for samples with corundum-blasted surfaces stored in cell medium.

## 1. Introduction

Total joint replacement (TJR) meets high quality and safety standards and has become a frequent surgical procedure in order to restore joint function [1]. However, implant revision remains a relevant problem in clinical use. Failure of TJR is mainly due to aseptic loosening caused by inflammatory reactions due to wear particles [2]. Postoperative implant-associated infections are rare but considered devastating complications after TJR. Although surgical techniques and environmental conditions during the surgical intervention have improved over the years, infections occur with a frequency of 0.5–2% with incisive consequences for the patients and medical costs [3]. Most implant-associated infections are caused by *Staphylococcus aureus* and *Staphylococcus epidermidis* [4, 5].

Immediately after implantation bacteria, and human host cells compete for the implant surface in the so-called “race for the surface” [6]. If bacteria adhere to the implant surface prior to human bone cells, biofilm formation might occur and osseous integration of the implant is precarious. In terms of biofilms, the treatment of implant-associated infections can be further hindered by the thus increased bacterial resistance against antibiotics [7]. Novel developments of ion-based antimicrobial implant surfaces such as silver (Ag) [8] or copper (Cu) [9] might offer a possible solution to this problem. Various *in vitro* and *in vivo* studies confirm the antibacterial properties and cytocompatibility of Cu [10–13]. Other alternative antibacterial materials and agents are in development or already in use to prevent or treat implant-associated infections [14–18]. *In vitro* investigations of the antibacterial effects are usually performed on simplified samples and under simplified testing conditions, whereas *in vivo* tests are usually closer to the final application. *In vitro* conditions are often adjusted according to the respective test, that is, cell biological and microbiological tests are performed with regard to their specific test protocols. Test fluids, storage times and fluid volume are essential parameters to characterise the antibacterial behaviour as well as the cytocompatibility and release kinetics of the antibacterial agents. Furthermore, the surface characteristics, such as the surface roughness, are an important aspect for coated surfaces with regard to the ion release properties [19]. Moreover, the ion release of ion-based antimicrobial coatings is of essential interest for the bactericidal activity and the cyto-compatibility of the coating.

The aim of the present study was to evaluate the Cu release characteristics of two different plasma surface treatments doped with Cu according to the storage fluid and surface roughness of the samples in order to provide an approach for possible standardised investigations in the future.

## 2. Materials and Methods

Titanium alloy (Ti6Al4V) discs (11 mm in diameter, 2 mm in height) were used as specimens for the investigation of ion release characteristics of Cu-doped plasma implant treatments. Two different plasma processes applying Cu were used: a plasma immersion ion implantation process (Cu-PIII) [20] and a pulsed magnetron sputtering process of a Ti-Cu film [21].

To analyse the influence of the composition of the test fluid on the Cu release, the Cu-PIII, and Ti-Cu-coated test samples were placed in 24-well plates and covered with 700 *μ*L test fluid. Additionally, uncoated Ti6Al4V test samples were submersed as a reference. The following test fluids were used: double-distilled water (TKA Wasseraufbereitungssysteme GmbH, Niederelbert, Germany), human serum (invitrogen, Darmstadt, Germany), and Dulbecco's Modified Eagle's medium (DMEM, Invitrogen, Carlsbad, USA) with 10% fetal calf serum (FCS Gold, PAA Laboratories GmbH, Pasching, Austria) as well as 1% gentamicin (Ratiopharm GmbH, Ulm, Germany). Subsequently, the samples were incubated at 37°C in a humidified atmosphere with 5% CO_2_ for 24 h to simulate physiological conditions. All samples were corundum-blasted before plasma treating and exhibited a surface roughness after plasma treatment of Ra = 3.76 ± 0.7 *μ*m and 2.02 ± 0.1 *μ*m for the Cu-PIII and Ti-Cu coatings, respectively. Three samples of each coating were submersed for each test fluid configuration.

In addition, to evaluate a possible influence on the Cu release due to surface topology and roughness, three different surface treatments of the Ti6Al4V samples were performed before Cu-PIII plasma treatment: polishing (Ra = 0.09 ± 0.09 *μ*m), hydroxyapatite (HA) blasting (Ra = 1.17 ± 0.2 *μ*m), and corundum blasting (Ra = 4.44 ± 0.5 *μ*m). Subsequently three Cu-PIII treated samples of each surface roughness, were immersed in double-distilled water for 5 days. Furthermore, roughness was investigated after storage in DMEM for 24 hours.

After the lapse of submersion time, the supernatants were removed from the samples and 1% nitric acid (HNO_3_) was added to stabilize the released Cu ions. In addition, the supernatants were diluted for the following atomic absorption spectrometry (AAS) analysis. By means of an AAS with electrothermal atomization (ZEEnit 650, Analytik Jena AG, Jena, Germany), the concentration of Cu ions released into the supernatants from the Cu plasma treatments was determined.

Thereby, the solutions of the different samples were evaporated in a three-step process (90°C for 20 s, 105°C for 20 s, 110°C for 10 s) followed by a pyrolysis phase at 850°C (10 s) in a platform tube. The pyrolysis phase eliminates residual organic material and combusts solid particles from the solution into ash. Using a rapid heat increase (1500°C/s), the tube was heated to 2000°C for 4 s to vaporize and convert solid particles into free atoms. This step also included the element analysis using a hollow cathode lamp with a Cu cathode radiating at 324.8 nm. Parts of the total emitted intensity were absorbed by the Cu atoms present in the tube from the diluted solution samples of the release experiments. The measured intensity was compared with the intensity of a standard Cu reference allowing the determination of the Cu concentration in the supernatants. In a final step the platform tube was cleaned by heating up to 2300°C for 4 s.

All data were stored and analyzed using the SPSS statistical package 15.0 (SPSS Inc. Chicago, Ill, USA). Descriptive statistics were computed for continuous and categorical variables [22]. The statistical data included mean and standard deviations of continuous variables, frequencies, and relative frequencies of categorical factors. Comparisons within the independent groups were achieved using the ANOVA test (Post Hoc LSD). All *P* values resulted from two-sided statistical tests, and values of *P* < 0.05 were considered to be significant.

## 3. Results

**Figure 1:**
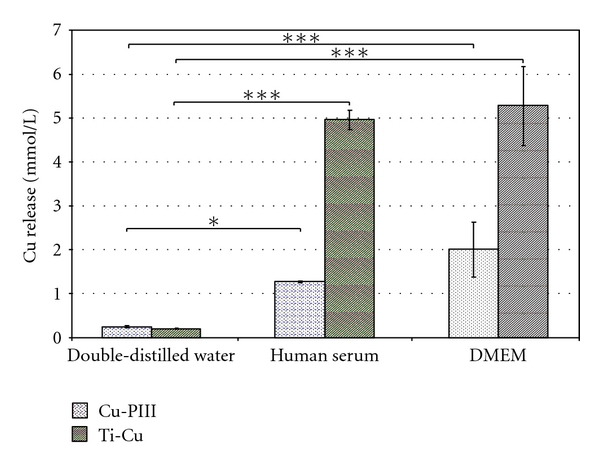
Copper concentration of Cu-PIII- and Ti-Cu-coated corundum-blasted Ti6Al4V surfaces submersed in 700 *μ*L of different supernatants (human serum, double-distilled water, and DMEM) for 24 h at 37°C and 5% CO_2_; ANOVA (Post Hoc LSD) test, *P < 0.05, ***P ≤ 0.001.

The uncoated Ti6Al4V control test samples showed no traces of copper in the supernatant. However, specific release characteristics were found for the analysed Cu-doped Ti6Al4V samples in different supernatants (Figure 1). Hereby, DMEM provoked the highest Cu ion release with a significant increase compared to double-distilled water (*P* ≤ 0.001), but no statistical significance was observed when compared to human serum (*P* ≥ 0.068). Higher Cu concentrations were released from the Ti-Cu films than from the Cu-PIII-treated surfaces in human serum (4.96 ± 0.22 mmol/l versus 1.25 ± 0.01 mmol/l) and in DMEM (5.27 ± 0.90 mmol/l versus 2.00 ± 0.63 mmol/l), respectively. Using double-distilled water, the observed concentration of released Cu was significantly lower (*P* ≤ 0.019) compared to human serum and DMEM and approximately the same for all samples with different Cu treatments (Ti-Cu: 0.20 ± 0.01 mmol/l, Cu-PIII: 0.25 ± 0.02 mmol/l).

**Figure 2:**
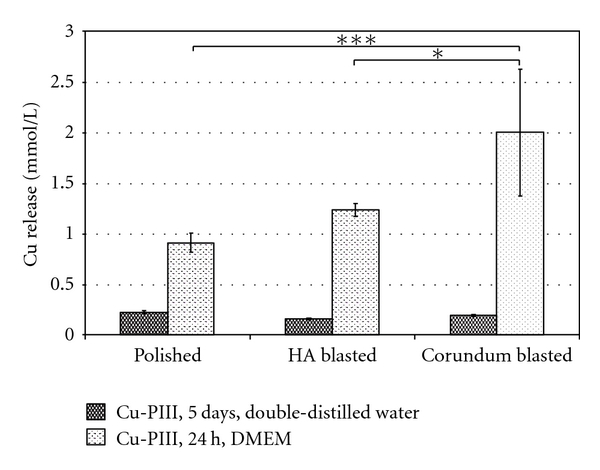
Copper concentration of Cu-PIII-treated Ti6Al4V samples with varying substrate surface roughness (polished, HA blasted, corundum blasted), submersed for 5 days in double-distilled water and 24 h in DMEM at 37°C and 5% CO_2_. ANOVA (Post Hoc LSD) test, *P < 0.05, ***P ≤ 0.001.

The surface roughness did not reveal a significant influence on the Cu release in double-distilled water (Figure 2). Polished surfaces resulted in a Cu concentration in the supernatant of 0.23 ± 0.01 mmol/l. For the HA and corundum-blasted surfaces the Cu concentration is in the same dimension at 0.16 ± 0.01 mmol/l and 0.20 ± 0.01 mmol/l, respectively. In comparison to DMEM, the Cu concentrations were significantly lower (*P* ≤ 0.007) for all surface topologies using double-distilled water. Corundum-blasted samples submersed in DMEM showed the highest Cu levels in the supernatant (2.00 ± 0.63 mmol/l, *P* ≤ 0.004). A decrease in Cu concentration was observed for the polished samples in comparison to the HA-blasted (*P* = 0.156) and corundum-blasted (*P* ≤ 0.004) samples.

## 4. Discussion

In order to enhance implant survival, bioactive coatings have moved into the focus of research and development. Due to the increasing risk of implant infections from multiresistant bacteria such as MRSA (multiresistant *Staphylococcus aureus*) [10, 11, 23] or ESBL (enterobacteria producing extended spectrum beta-lactamases) [24], different antimicrobial coatings [17, 25–28] are being developed. However, the mechanical, biological and chemical properties of such innovative coatings have to be investigated thoroughly. Cu ions can be an effective antimicrobial agent to inhibit bacterial growth and biofilm formation on endoprosthetic surfaces [10, 29]. Analyses of the ion release are strictly necessary for the determination of relevant Cu concentrations required in order to regulate both antimicrobial effects and compatibility to human cells. Furthermore, the release kinetics of copper-doped surfaces needs to be investigated in standardised tests in order to ensure effective and valid ion concentrations. In this context, however, standardised test conditions have not been established so far.

The AAS is a suitable device to measure Cu concentrations in supernatants. Uncoated samples did not show any Cu in any of the analysed supernatants, whereas the Cu-treated samples revealed differences in Cu concentration. Nevertheless, using an AAS, both Cu^+^ and Cu^2+^ ions are assessed at the same time without any distinction. However, only Cu^2+^ ions cause an antimicrobial effect [30]. Hence, the concentration alone is not enough to predict the effectiveness of the coating and should be supported by microbiological tests.

Investigations of the ion release of an antimicrobial coating should coincide with cell biological tests using human cells to study biocompatibility of the coating. Furthermore, test fluid volumes should be the same for all studies, ensuring similar Cu concentrations. A test fluid volume of 700 *μ*L was chosen to represent *in vivo* conditions. After conventional implantation technique, Wu et al. [31] observed that 40% of an uncemented femoral stem showed no bone contact with an average gap width between the bone and the femoral stem of 0.77 mm. In relation to the test samples deployed in this study, a volume of approximately 200 *μ*L would be adequate to simulate the gap volume at the uncemented stem. However, 200 *μ*L is not enough to cover the test samples completely, which would make *in vitro* testing impossible. Therefore, a volume of 700 *μ*L was chosen as a compromise, which was the smallest possible volume that assured proper cell and bacterial growth in the supernatant.

The results of this study show that the amount of Cu ions released into the supernatant depends on various factors. The supernatant and its properties to dissolve Cu ions plays an important role. Surprisingly, the Cu concentration in double-distilled water for the Cu-PIII coating after 24 hours did not differ from the Cu concentration after 5 days. Therefore, double-distilled water showed an early Cu saturation for both tested coatings, whereas human serum and DMEM revealed a much higher Cu concentration in the supernatant. In fact, the highest concentration was obtained in DMEM for the Ti-Cu coating. The presence of serum proteins increases the solubility of Cu in the supernatants significantly compared to the double-distilled water because of the complex compounds between copper and amino groups found in the proteins in human serum and DMEM. Wu et al. [32] showed a higher concentration of Ag^+^ ions after 24 h in cell culture medium as in simulated body fluid, which coincides with the Cu concentrations found in our present study. However, based on the current results, it is not possible to appoint a Cu ion saturation level for neither human serum nor DMEM. Repeated and cumulative investigations at different time periods need to be carried out and could provide a precise saturation level.

Surface roughness increases the surface area in contact with the supernatant. With rising surface roughness, an increase of the Cu concentration in the supernatant was observed. Compared to a polished surface, the corundum-blasted surface released approximately three times as many Cu ions within 24 h. In double-distilled water, the Cu concentration levels remained constant after 5 days regardless of the surface roughness, since the Cu saturation has set in within the first 24 h.

Temperature may also influence the ion release characteristic of an implant material. Therefore, the release studies should be carried out at 37°C body temperature. A time-dependant Cu release behaviour, cumulative and non cumulative, of the analysed coatings is currently under investigation. First results show that most of the Cu is released during the first 24 h, followed by a highly reduced release rate during the succeeding days. Furthermore, the effect of copper ions on human cells and tissue is currently under investigation provided by cell biological and microbiological tests as well as animal studies using an infection model.

## 5. Conclusion

When testing antimicrobial Cu-coated implant surfaces, it is important to apply appropriate test conditions regarding the ion release into the surrounding tissue. With respect to future clinical applications of the coated implants, a suitable test fluid such as human serum or DMEM should be used to coincide with cell biological and microbiological studies; otherwise, false conclusions may be drawn. Furthermore, it is important to use test samples with adequate surface properties close to those needed in the final application, since the surface roughness can affect the ion release dramatically.
